# Investigation on up-flow anaerobic sludge fixed film (UASFF) reactor for treating low-strength bilge water of Caspian Sea ships

**DOI:** 10.1186/s40201-015-0181-3

**Published:** 2015-03-20

**Authors:** Seyyed Mohammad Emadian, Mostafa Rahimnejad, Morteza Hosseini, Behnam Khoshandam

**Affiliations:** Department of Chemical Engineering, Semnan University, Semnan, Iran; Department of Chemical Engineering, Babol University of Technology, Babol, Iran

**Keywords:** Anaerobic treatment, UASFF reactor, COD, pH, TSS, Oil content

## Abstract

**Background:**

In order to meet the International Maritime Organization (IMO) objectives, the main purpose of this study was using the cheap and practical wastewater treatment system for low-strength bilge water of Caspian Sea ships; therefore, the low-strength bilge water of the Caspian Sea ships has been treated by up-flow anaerobic sludge fixed film (UASFF) reactor at the ambient temperature.

**Results:**

The reactor operated at two hydraulic retention times (HRTs) of 10 h and 8 h. The organic loading rates (OLR) ranged (0.12-0.6) g chemical oxygen demand (COD)/l.day. At the beginning of the experimental procedure, the sludge was immobilized on the surface of the support materials. After 10 days of batch feeding of the reactor with the wastewater as an acclimation period (with COD removal of 59%), the reactor operated continuously. At the end of the experiment, with the HRT of 8 h and OLR of 0.6 g COD/l.day, the COD and total suspended solid (TSS) removal efficiencies reached the amounts of 75% and 99%, respectively. In addition to the good features of the reactor in removing COD and TSS, the effluent oil concentration was significantly lower than the standard value (15 ppm) which has been laid down for the discharge of the bilge water from ships by the IMO.

**Conclusions:**

The obtained data demonstrated that UASFF reactor is an appropriate system for treatment of a low-strength bilge water.

## Background

Three kinds of wastewater exist which are produced on ships: black water, grey water and bilge water. Bilge water is the mixture of water, oily fluids, lubricants, cleaning fluids and other similar wastes that accumulate in the lowest part of a ship. The International Maritime Organization (IMO) regulations necessitate that any oil and oil residue discharged in wastewater streams must contain less than 15 mg/l of oil [1]. The common technology is used in ships for treating bilge water is oil water separator (OWS) using the buoyancy difference of oil and water for separation. Cleaning agents in bilge water can create an emulsion of oil in water. When emulsification takes place, buoyancy difference of oil and water is too small to be treated properly via the existing OWS technology.

Other techniques have been studied in order to treat bilge water including membrane technology [2,3], electrocoagulation [4,5], UF/photocatalytic oxidation [6]. Some disadvantages were reported associated with the application of membrane in treatment of bilge water such as: their relatively high cost of production because of the expensive raw materials, fouling which has a number of negative effects such as the reduction in membrane flux, additional capital and maintenance cost due to membrane replacement and regeneration [2,7]. Karakulski et al. reported a promising usage of laboratory-scale ultrafiltration pilot plant with tubular membranes for the treatment of bilge water. However, the use of additional photocatalytic oxidation stage was necessary to eliminate the residual oil [6]. Rincon et al. concluded that the electrocoagulation process was an effective method in destabilization of oil in water emulsions and removing of heavy metals. However, the electricity consumption and the use of additional flotation method should be considered for improving the treatment efficiency [5].

Anaerobic treatment is a well-established technology for treatment of wastes and wastewaters because it is technologically simple for low energy consumption and it is an efficient, economical and environmentally-friendly method. The final product of anaerobic digestion is biogas which is a mixture of methane and carbon dioxide. These produced components can be applied for heating and upgrading natural gas quality or co-generation [8]. One of the most notable developments in anaerobic treatment process technology is the up-flow anaerobic sludge blanket (UASB) reactor. The UASB reactor has some positive features, such as short hydraulic retention time that allows high organic loadings. Furthermore, it has a low energy demand and area requirement [9,10]. A major problem of UASB reactor is the long period (several months) required for the formation of granule sludge in the reactor [11]. Although formation of granule in UASB reactors has some advantages, successful treatment of wastewaters with flocculent sludge UASB reactors have been reported [12,13]. The up-flow anaerobic sludge fixed film (UASFF) reactor configuration has combined the advantages of both UASB and Up-flow anaerobic fixed film (UAFF) reactors. This kind of reactor is efficient in the treatment of dilute to high strength wastewaters at low to high Organic Loading Rates [14,15]. The packing medium in the hybrid reactor plays an important role in giving a better performance to the UASB reactor such as increasing solids retention by dampening short circuiting, improving gas/liquid/solid separation, and providing surface for biomass attachment.

Bilge water is classified as the low strength group of wastewater [14]. Although anaerobic process is used for the treatment of medium and high strength wastewaters, it has already been applied successfully for a number of waste streams including low strength wastewaters [16-18].

In this study, the efficiency of UASFF reactor (on the basis of COD, TSS, oil removal and biogas production) has been studied in treatment of low-strength bilge water under different low organic loading rates at the ambient temperature.

## Methods

### Experimental system

**Figure 1:**
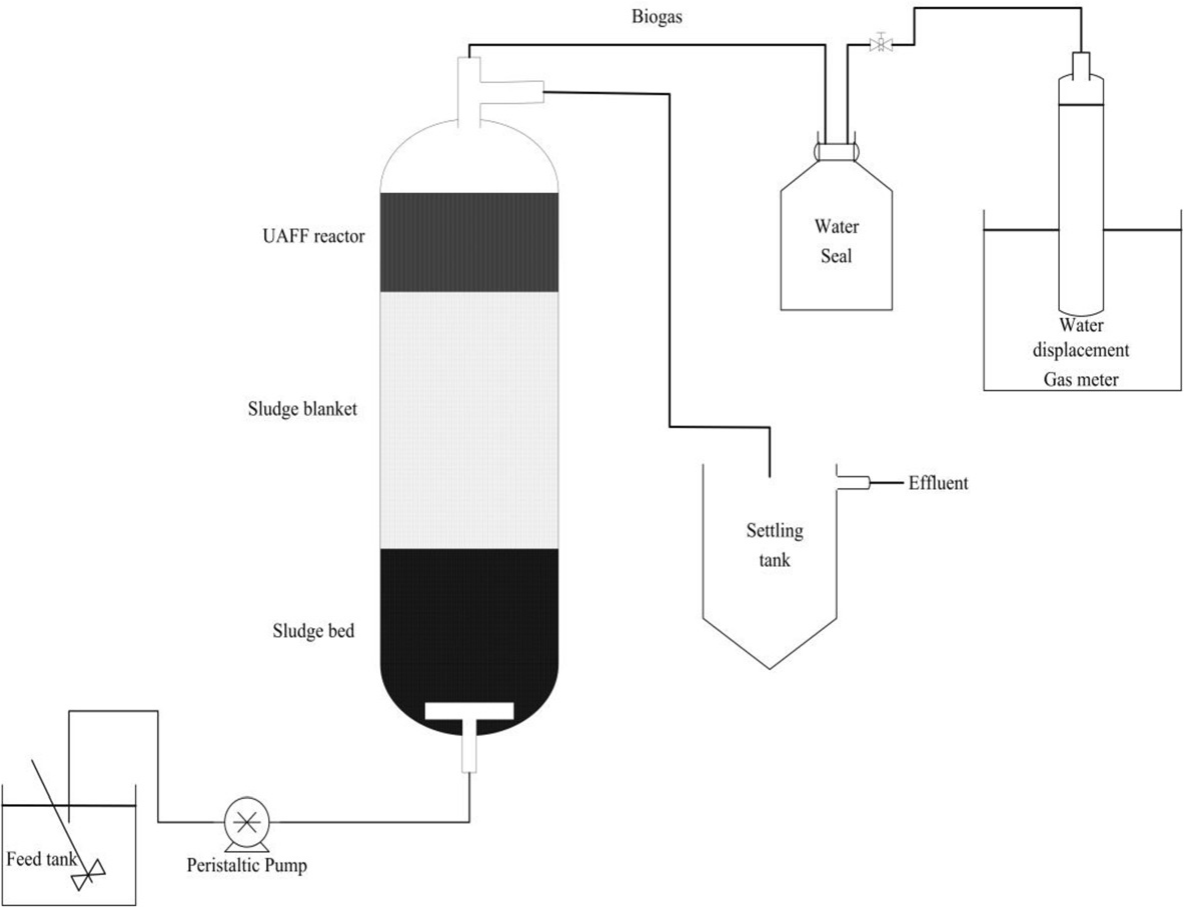
**Schematic diagram of the used experimental setup in this research.**

The schematic diagram of the laboratory-scale UASFF reactor used in this study is presented in Figure 1. The fabricated Plexiglas bioreactor column had an internal diameter of 4.4 cm and a liquid height of 194 cm. The column consisted of three sections including bottom, middle and top. The bottom part of the column, with a volume of 1823 ml operated as a UASB reactor whereas the middle part of the column with a volume of 855 ml was used as a fixed film reactor. The top part of the bioreactor with a volume of 273 ml was an unpacked column prior to the effluent overflow. The fixed film section of the column was randomly packed with 270 billowy pieces of PVC rings with diameter of 15 mm and the height of 13 mm (150 m^2^/m^3^ specific surface areas for each one). The media in the reactor were stabled by using a plastic mesh. The wastewater as a substrate was continuously fed to the base of the reactor, under the bed of active sludge, through a T-inlet connected to a peristaltic pump. An outlet was provided at the top of the reactor that was connected to a 1 liter funnel shaped settling compartment served as a sedimentation part where the final effluent was collected from the top of this tank. The effluent tube was connected to a gas tank for gas collection by water displacement whenever wanted to measure the produced biogas volume. The reactor operated at ambient temperature (15–25)°C.

### Wastewater characteristics

The bilge water was collected from a tank with which anchored cargo ships typically discharged their bilge water to it at Amirabad port, Behshahr, Mazandaran, Iran. The samples were collected from the top, middle and the bottom of the tank in order to provide a uniform sample from all parts of the tank. The UASFF reactor was fed with bilge water pre-settled for 10 min. the characteristics of pre-settled bilge water are summarized in Table. 1. The pH of the feed was adjusted to 6.8 to 7.2 by adding diluted HCl. The only supplementary nutrient, MgNO_3_ as a nitrogen supply, was added to yield a COD: N ratio of 250:5.

**Table 1:** **characteristics of pre-settled bilge water; TN and TP were measured in COD = 50 mg/l**

**Parameter**	**Value**
PH	8 – 9
COD (mg/l)	20 – 200
TS (mg/l)	800 – 2400
TSS (mg/l)	220 – 1760
TN (μg/l)	836
TP (μg/l)	211

### Inoculum (seed sludge)

The reactor was seeded with a mixture of activated sludge from the aerobic wastewater treatment of the Mazandaran pulp and paper industry and a non-granular sludge obtained from an up-flow anaerobic sludge blanket reactor operating with cheese whey wastewater from the Gela food industry of Amol, Mazandaran, Iran. The TSS of the mixture was 13 g/l. The non-granular sludge was methanogenically active as the biogas bubbles were apparently observed stripping from the sample surface which was collected in a closed bottle.

### Analytical methods

Several monitoring parameters were evaluated during the entire operation, including COD, TSS and oil concentrations, as well as pH, temperature and biogas production volume rate. For COD analysis, HACH’s Method 8000, a combination of reactor digestion method and colorimetric method, was used [19]. This method is equivalent to standard method 5220D: closed reflux, colorimetric method [20]. Analytical determination of TSS was carried out in agreement with the standard methods for the examination of water and wastewater [20]. Analysis of oil was determined according to USEPA Method 1664, N-Hexane gravimetric method. Temperature and pH were measured using a pH/temperature probe (HANNA, PH212, Germany) with automatic temperature compensation. The method used in pH measurement was generally in compliance with standard method 4500B [20]. Biogas was collected by water displacement and the volume was read from a calibrated gas collection cylinder.

### Start-up and operation scheme

Start-up period usually takes a long time. In order to decrease this time, the immobilization of biomass on the support material was done. So, the mentioned mixture of sludge was used by means of a technique described by Zaiat et al. [21]. The support material in combination with the sludge was stored in 1.5 l closed bottle and homogenized for the period of a week by using a shaker so as to secure steadier immobilization of bio-particles in the supporting material. It is noticeable that this initial immobilization of biomass in the support materials has never been done by the other authors. After this stage, the packing material was filled in its place in the UASFF reactor.

The reactor was inoculated with 500 ml of the same sludge mixture. In order to acclimatize the sludge with bilge water, the reactor was daily batch feed with the bilge water (50 mg/l) for 10 days. After each feed, the liquid content of the reactor was continuously circulated for 1 day (until the next feed). The acclimation period permitted oxygen level decrease to prevent inhibition of anaerobic bacteria as well as the bacteria population to adjust with the feed wastewater. The TSS concentration of the sludge after the 10-day batch-fed period was 16.5 g/l. A COD removal of about 59% was achieved at the end of this acclimation period.

**Figure 2:**
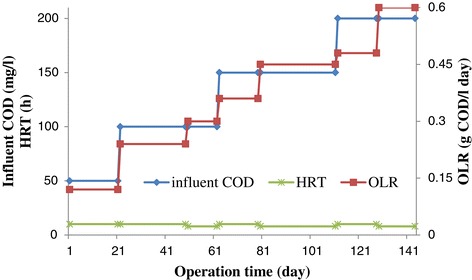
**Start-up and operation scheme for UASFF reactor.**

The purpose of the start-up of anaerobic bioreactors is to grow, build up and retain a sufficient concentration of active and well balanced biomass. The start-up was carried out by using stepped organic loading to produce the most rapid biomass development. The start-up stage of the process was began by continuous feeding of the reactor with an initial influent COD concentration of 50 mg/l, HRT of 10 h and consequently organic loading rate of 0.12 g COD/l day which is remarkably a low value. This influent COD concentration was applied for 21 days. After that, it is increased to 100 mg/l from the day of 21 to 49. The HRT of 10 h was kept constant throughout the start-up duration. The reactor was allowed to reach steady state condition before each OLR change. When effluent COD reached a relatively constant value, the steady state condition was achieved and then influent OLR can be raised [22]. The experimental procedure is illustrated in Figure 2.

During the experiment, COD reduction, pH and biogas production were monitored daily. The TSS reduction was usually measured every other day. Also oil reduction was checked 2 times throughout the experiment. The first check was after the end of the start-up period and the second check was after the completion of the whole experiment.

## Results and discussion

### Bioreactor start-up

#### pH

**Figure 3:**
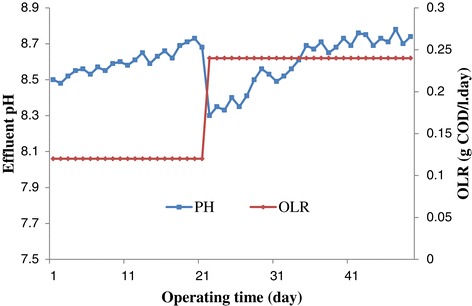
**Change of PH during start-up.**

Changes in acidity (pH) of the effluent from the UASFF reactor during the start-up stage is shown in Figure 3. The pH was comparatively stable (varying from 8.3 to 8.78), which was suitable for efficient methanogenesis, indicating that the system had sufficient alkalinity to neutralize organic acids coming from the hydrolysis and fermentation stage [23]. After 22 days, a sudden decrease in pH from 8.68 to 8.3 took place which is attributed to the accumulation of the produced VFA (Volatile Fatty Acid) because of enhancement of the OLR. Accumulation of VFA in the reactor did not sour the reactor. The similar result was reported by Van Haandel and Lettinga about the treatment of domestic wastewater [24].

### COD removal efficiency

**Figure 4:**
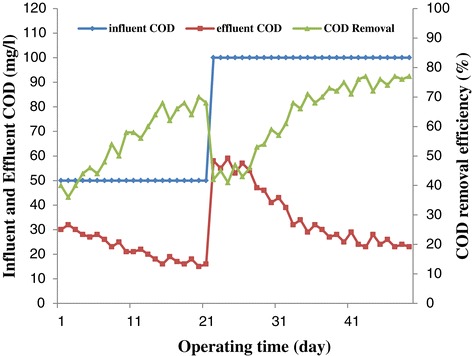
**Bioreactor performance during start-up period.**

The bioreactor performance during the start-up is shown in Figure 4. The reactor was fed with an influent COD of 50 mg/l and the COD removal efficiency was increased from 40% to 68% in the first 21 days. Subsequently, the influent COD concentration was enhanced to 100 mg/l for the remaining 28 days of the start-up period. As the graph shows, increase in influent COD from 50 mg/l to 100 mg/l caused a decline in the COD removal efficiency from 68% to fewer than 42% which can be attributed to the fact that the system was put under stress. This phenomenon can be due to the increase in VFAs concentration which is recognizable from sudden decrease in effluent pH at day 22. Similar observation was reported by other authors [23,25,26]. The system recovered shortly and adapted to the new condition with time. Though, in terms of removal efficiency, the increase in influent COD from 50 mg/l to 100 mg/l led to an increment in terms of the COD removal efficiency from 68% to 77%, implying that the sludge was acclimated appropriately to the bilge water. A comparison between Figures 2 and 3 shows a similar trend between effluent pH and COD removal efficiency which concurs with the results obtained by Zhang et al. [23].

### Biogas production rate

**Figure 5:**
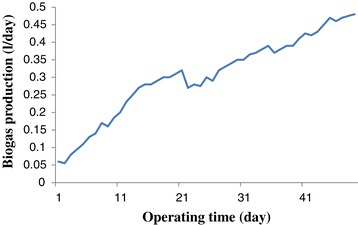
**Biogas production rate during start-up period.**

The biogas production rate along the start-up is shown in Figure 5. As the profile shows, the biogas volume rate increased from 0.06 l/day to 0.37 l/day in the first 21 days. The introduction of higher COD to the reactor was caused a sudden decrease in biogas production at day 22. The excess VFA which was produced at this time inhibited the methanogenic bacteria from their efficient performance and as a consequence, the biogas production decreased [27,28]. However, the biogas production increased again from day 26 and this indicated that the microorganisms acclimated to the new condition. At the end of the start-up period, the biogas production reached an amount of 0.48 l/day. During the start-up, the biogas production raised like the COD removal efficiency which was in agreement with another author’s result [28]. The reason for the increased biogas production is due to proper anaerobic population development [26].

The overall performance of the reactor during the startup was satisfactory. It is known that the selection of seed material plays a crucial role in minimizing the time required for start-up duration [26]. In addition, it is clearly understood that the initial immobilization of microorganisms on the surface of the support materials had a key role in progressing the start-up procedure.

### Later operation stage

After a 49-days startup period, the reactor was operated at HRTs of 10 h and 8 h with three different influent COD concentrations (from 100 mg/l to 200 mg/l) to evaluate the effect of low organic loadings on the reactor performance.

### pH

**Figure 6:**
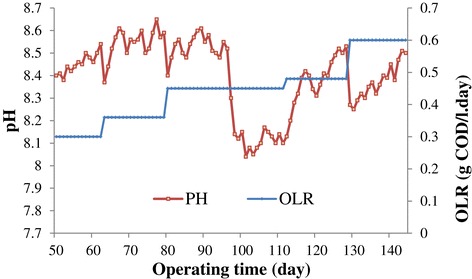
**Change of pH during later operation.**

Figure 6 shows the variation of effluent pH during the operation. As it shows, the pH of the treated wastewater was in the range of 8.04-8.61 which is indicative of the buffering capacity of the reactor. There was a sudden decrease in pH from 8.52 at the day of 96 to 8.1 at the day of 111 because at this stage of the operation, the effect of the nutrient was tested. For testing this effect, from the day of 96 to 101, the addition of the nutrient was ceased and after that the new nutrient, NH_4_Cl, was added to the reactor till the day of 111. Decrease in pH in this period of the operation proved that more VFA was accumulated in the reactor due to the lower activity of the methanogenic bacteria which was responsible for consuming of the VFA. However, the reactor recovered itself because of introducing MgNO_3_ as the nutrient to the reactor and pH increased again which is indicative of the increase in methanogenic bacteria functionality.

### COD and TSS removal efficiencies

**Figure 7:**
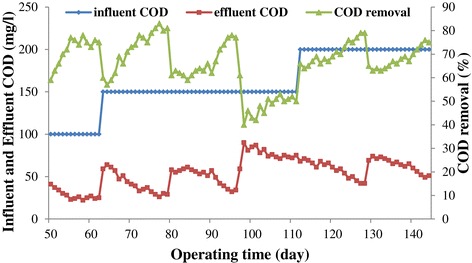
**Bioreactor performance during the later operation stage.**

**Figure 8:**
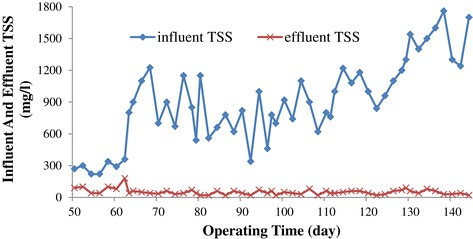
**TSS removal during operation.**

The performance of UASFF reactor based on COD and TSS removal efficiencies during the operation period is shown in Figure 7 and Figure 8, respectively. As illustrated in the Figure 7, the COD removal efficiency went through an increasing trend from a low amount of 59% to a maximum of 77% during the first 46 days except at the beginning of each OLR increment, there was a corresponding decrease in COD removal efficiency but the system recovered shortly and adapted to the new conditions with time like the start-up period [14,26]. As Figure 8 illustrates, the influent TSS concentration is unstable because of the poor agitation that was provided in feed tank. As the graph shows, the effluent TSS concentration was very low which is indicative of the good performance of the reactor in eliminating the suspended solids. As it was mentioned before, the effect of the nutrient on the performance of the reactor was tested during the days of 96 to 111. According to Figure 7, the COD removal efficiency decreased from 77% to 42% during the days 96 to 101,the period that the addition of MgNO_3_ was ceased to the reactor. After that, by addition of new nutrient (NH_4_Cl) to the reactor, the COD removal efficiency increased a little and reached an amount of 50% at day 111. The obtained data demonstrated that MgNO_3_ was a better choice than NH_4_Cl in the present study. Therefore,MgNO_3_ was introduced to the reactor again from day 111. Although the COD removal efficiency decreased considerably during the days of 96 to 111, the TSS removal efficiency was still as high as the other days of the operation (see Figure 8). For instance at day 98, the COD removal efficiency declined to the amount of 40% while the TSS removal efficiency was 97%. This phenomenon indicates that most of the COD removal during this study was due to the reduction of the soluble COD and not the suspended COD. By increasing the COD influent and introduction of MgNO_3_ as the nutrient to the reactor at the day of 111, the COD removal efficiency raised again and it reached an amount of the 75% at the end of the study. The obtained result is comparable with the COD removal efficiency achieved by Sun et al. in which they reached the percentages of 59% in treatment of synthetic bilge water by using an aerobic moving bed bio-reactor (MBBR) [29]. In addition, the reactor achieved TSS removal efficiency of 99% at the end of the experiment which was a noticeable result. The good performance of the reactor in the eliminating of the TSS content of the wastewater during the operation suggests that most proportion of the TSS removal is due to the entrapment and adsorption of the suspended solids at sludge bed and fixed film [30]. The TSS removal efficiency throughout the experiment did not differ significantly which is in agreement with Ligero et al. results who reported the TSS removal efficiency of an UASB reactor for all values of HRT was not very different [31].

### Biogas production rate

**Figure 9:**
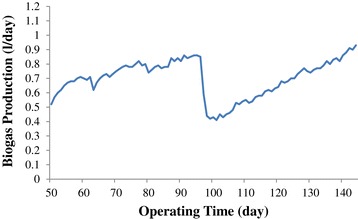
**Biogas production during later stage.**

As Figure 9 shows, the biogas production rate increased from 0.52 l/day at the day of 50 to 0.85 l/day at the day of 96. Lettinga reported that the reduction of BOD and COD contributed to the gas production [32]. One can see in Figure 9, the biogas production decreased from 0.85 l/day at the day of 96 to 0.41 l/day at day of 101 (without nutrient addition) and then it reached to the amount of 0.53 at the day of 111 (with addition of NH_4_Cl as the nutrient) which can explain that the activity of methanogenic bacteria decreased at this stage of the operation. By increasing the influent COD and the addition of MgNO_3_ as the nutrient at the day of 111, the biogas production increased again and it continued to the amount of 0.93 l/day at the end of the study.

### Oil content

**Figure 10:**
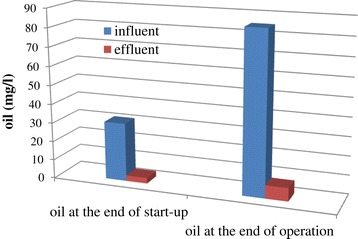
**Oil removal at two point of operation.**

The reduction of oil content of the wastewater at the end of start-up and operation of the reactor is shown in Figure 10. Presence of oil in wastewaters leads to the accumulation of it on the surface of the sludge which causes foaming and scum formation which eventually lowers the digestion efficiency [10]. There was no sign of foam and scum in the reactor which was indicative of the good performance of the reactor. As Figure 10 shows, either at the end of the start-up or at the end of the operation, the oil effluent concentration was below 15 mg/l which is IMO standard level for discharging the wastewater from ships [1]. The obtained result was so promising in comparison with the outcome of the Sun et al. [29]. They reported that the effluent oil content from the MBBR at the HRT of 8 h was about 30 mg/l which was about double times higher than the standard level of discharging [29].

### Sludge

The TSS concentration of the sludge in the reactor increased from 16.5 g/l at the beginning of the start-up to 67 g/l at the end of the study. This sludge production in the reactor may be attributed to (1) flocculation and entrapment of the non-biodegradable influent TSS, forming the inert sludge mass fraction and (2) the biological sludge mass that is generated as a result of anaerobic conversion in the hybrid reactor but because of the mentioned reasons in COD and TSS removal section, the entrapment of the suspended solids in the sludge seems to have more effect on increasing the TSS content of the reactor sludge. So, the sludge acted as a filter for removing the suspended solids from the wastewater [33]. Therefore, the UASB reactor had a noticeable effect on removing the TSS content of the wastewater [34-36]. At the end of this study, a flocculent sludge was observed without any granule formation in it. As the other authors reported, low strength wastewater can lead to substrate transfer limitation and cause inhibition of granulation or can make it difficult to maintain granules [37,38].

### Wastewater appearance

**Figure 11:**
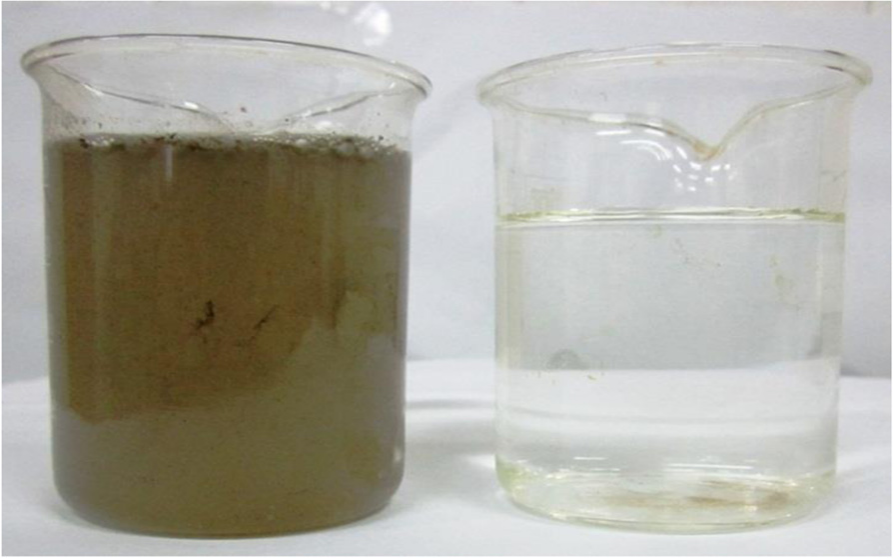
**Influent and effluent of the reactor at the end of the operation.**

Figure 11 illustrates the apparent difference between influent and effluent of the reactor at the end of the operation. As the Figure 11 shows, the reactor had a good performance in decolorizing of the wastewater which can be as another advantage of the reactor.

## Conclusions

In this study, anaerobic treatment of dilute bilge water was performed by using UASFF reactor at ambient temperature. After a good resulted immobilization of sludge in the support materials and start-up period, the COD and TSS removal efficiencies reached the amounts of 75% and 99% at the end of the operation, respectively. The results showed that the sludge blanket acted as a filter for removing the suspended solids from the wastewater and the major proportion of COD removal was due to the soluble and not suspended COD. The biogas production rate reached an amount of 0.93 l/day at the end of the experiment and effluent oil concentration is remarkably below the standard amount which has been set by the IMO (15 ppm). The good performance of the bioreactor on appearance of the wastewater can be considered as another advantage of this type of the UASFF reactor. The immobilization of the biomass in the support materials had an important role in reducing the influent COD because they created a good media for methanogenic bacteria on their surface. According to the obtained results, it can be concluded that the UASFF reactor is a very promising option for the treatment of the low-strength bilge water, produced from the ships in Caspian Sea, at the ambient temperatures for implementation on the ships in a large scale.
